# “They think surgery is just a quick fix”

**DOI:** 10.3402/qhw.v9.24378

**Published:** 2014-07-11

**Authors:** Karen Synne Groven

**Affiliations:** 1 Institute of Health and Society, University of Oslo, Oslo, Norway; 2 Institute of Physiotherapy, Oslo and Akershus University College of Applied Sciences, Oslo, Norway

**Keywords:** Gastric bypass surgery, qualitative study, women, phenomenology

## Abstract

**Background:**

To prevent weight regain, patients undergoing weight loss surgery are encouraged to change their exercise and dietary habits. Building on previous research, the aim of this study was to explore women's experiences of changing exercise habits – focusing on women participating in a group based rehabilitation program including surgical as well as non-surgical participants.

**Findings:**

Based on interviews with the 11 women included in this study, as well as participant observation, two themes were identified; *1) Pushing ones tolerance limits*, and *2) Rebutting the “quick fix” fallacy*. Taken together, the findings showcase how being a part of this mixed group involved having to relate to social stigmas, as well as notions regarding successful and non-successful surgical outcomes. Although such notions may be useful in identifying potential challenges related to changing exercise habits, they do not illuminate the complexity of undergoing such changes following weight loss surgery.

**Conclusion:**

The findings point to the need of acknowledging patients' own exceriences to determine how successful they are after surgery. Given the findings, I argue for the need to reconsider the notion of success in relation to group based interventions.

*They think surgery is just a quick fix and then you are slim. But that's not how it works*.

This is Lilly, a 47-year-old woman who had lost more than 30 kg following weight loss surgery. Prior to her surgery Lilly felt too heavy to move and exercise in the manner she would have liked. However, during our interview, she repeatedly emphasized that attaining a slimmer and lighter body was not obtained by the surgery itself. It was, as she put it “by no means a quick fix.”

This was not the first time I was made aware of the popular conception that weight loss is automatic and easy following surgery. I was introduced to the phrase in 2005. As participant observer in a lifestyle program I noticed that one of the women kept mostly to herself. This aloofness aroused my curiosity; all of the other participants engaged in small talk during these sessions. When I happened to mention her to some of the other participants, they were quick to tell me that she had undergone weight loss surgery. They also told me that she had regained most of the weight and suggested this was evidence that taking a “quick fix” rarely paid off. Their perspective led me to wonder how women who chose to undergo this surgery experienced having to relate to popular assumptions concerning weight loss surgery as a quick fix while at the same time participating in group-based rehabilitation. While pondering these questions, I found myself discussing weight loss surgery with colleagues and friends. Several informed me that they knew or had heard of people—always women—whose life had dramatically improved following weight loss surgery. Some, however, had also heard of women who had suffered from major side effects after surgery, affecting their efforts to change lifestyle following the surgery.

The significance of lifestyle changes following weight loss surgery is increasingly emphasized in medical research. Although most individuals report dramatic weight loss during their first year after surgery, recent studies have reported variation in long-term results. By 3–5 years after the surgery, many patients report that they have regained some of the weight, whereas others report having regained significant amounts of weight (Hsu et al., 1998; Magro et al., 2008; Wadden et al., 2007). Such results have led to a rapid proliferation of lifestyle interventions in which persons undergoing weight loss surgery are increasingly expected to engage. By focusing on the importance of changing habits in terms of diet and exercise, such interventions are regarded as paramount to prevent weight regain in the long run (Colles, Dixon, & O'Brien, 2008; Hofsø et al., 2011; Sjöström et al., 2010).^1^

In their review article of 13 studies focusing on exercise and weight loss outcomes, Livhits and colleagues found a positive association between physical activity and post-operative weight loss. Furthermore, their meta-analysis of three studies revealed a significant increase in 1-year post-operative weight loss among individuals who exercised on a regular basis after surgery (Livhits et al., 2010). By comparison, Bond and colleagues found that participants could be divided into different categories based on their pre- and 1-year post-operative activity level. Although most participants remained either active or inactive, roughly one-third progressed from inactive to active, whereas 5% shifted from active to inactive (Bond et al., 2010). Taken together, these findings suggest that participants can be divided into three groups; those who report increasing their activity level following surgery, those who do not report any change, and, those who report becoming less active. However, these studies do not provide insight into patients’ experiences of being active following surgery as well as why some patients become more active whereas others become less active. Explicitly taking patients own experiences into consideration could provide greater nuance in a field hitherto dominated by quantitative “effect” studies (Engström & Forsberg, 2011; Warholm, Øien, & Råheim, 2014).

The most common bariatric procedure performed by surgeons in the United States as well as Scandinavia is the gastric bypass procedure (Buchwald & Williams, 2004). The gastric bypass as most commonly done today involves dividing the patient's stomach into two sections, consisting of a smaller pouch (30–50 ml) and a larger lower section. Additionally, 1.5 meters of the patient's intestine is disconnected. A section of the patient's intestine is then re-routed to the smaller upper pouch, thereby bypassing the larger stomach section and the volume of food it can potentially hold (Aasheim et al., 2007; Buchwald & Williams, 2004). Hence, the procedure is irreversible, providing a physical limitation to the amount the patient can eat, as well as reducing food absorption after ingestion resulting in more rapid and dramatic weight loss compared to other bariatric procedures. However, there are also various side effects associated with this procedure, including malnutrition, and various intestinal and digestive problems (Buchwald & Williams, 2004; Engström & Forsberg, 2011; Groven, Råheim, & Engelsrud, 2010; Maggard et al., 2005).

Both the risk of regaining weight and the side effects following gastric bypass surgery have gradually been documented in research. However, women's experiences of physical activity and exercise following this surgery have been a neglected field of research. “In a previous study, my co-authors and I have explored how gastric bypassed women experienced the interval aspect of group-based rehabilitation. A major finding in our study, was that the program represented a turning point in terms of getting started with training and physical activity on a daily basis. However, we also found that the women's changing habits were intimately intertwined with their changed and changing bodies, including side-effects and changes in their bodily capacity (Groven, Råheim & Engelsrud, 2013a).” Building on these findings, my ambition is to further explore and problematize the relational aspects of participating in a mixed group setting, including surgical as well as non-surgical participants. I address the following question:

*“How do women experience group-based training following gastric bypass surgery?”*

## Theoretical framework

In Merleau-Ponty's phenomenology of the body, perception and movement are intimately intertwined. Walking in the woods with a group of friends, for example, the perceiver will position her gaze and ears in ways that enable her to see and hear them. Our perception is in other words selective; something or someone is likely to emerge in the foreground of our attention whereas something or someone else recedes in the background of our attention. To put it in Merleau-Pontyan terms: It is a figure perceived against a background, an object or a person perceived in context (pp. 200–250).

The example from walking in the woods illuminates how perception is also a relational process in which the persons are directed towards the surroundings and the surroundings are directed towards the persons. Merleau-Ponty has this to say on the matter:

But we have learned in individual perception not to conceive our perspective views as independent of each other; we know that they slip into each other and are brought together finally in the thing. In reality, the other is not shut up inside my perspective of the world, because this perspective itself has not definite limits, because it slips spontaneously into the others and because both are brought together in the one single world. (Merleau-Ponty, 2002, p. 411)

There are, as revealed in this extract, some blurred boundaries between the perceiver and the perceived. As part of a common world, their experiences cannot be separated from each other, nor are they precisely the same. In his unfinished manuscript, *The Visible and the Invisible*, Merleau-Ponty sought new ways to elaborate on this relation. Through the notion of *flesh*, for example, he provided some illuminating images of how perceiver and perceived, body and world, and body and things are intertwined. In the following extract, he elaborates on this intertwining taking as his starting point the visibility of a woman's body:

A woman feels her body desired and looked at by imperceptible signs, and without even herself looking at those who look at her. The “telepathy” here is due to the fact that she anticipates the other's effective perception …. One feels oneself looked at not because something passes from the look to our body to burn it at the point seen, but because to feel one's body is also to feel its aspect for the other. (Merleau-Ponty, 1968, p. 245)

This extract highlights how the gaze of the other is an inter-subjective process involving both the perceiver's presence and the perceived person's previous experiences of being looked at and desired. Previous experiences intertwine with the situation here and now, shaping the person's sense of herself as well as her experiences of various activities and situations.

This intertwinement between body and world will be of relevance when exploring women's experiences of group-based training following gastric bypass surgery. When a woman loses a considerable amount of weight, she is likely to experience herself—as well as likely to be experienced by others—in new ways.

American physician and philosopher Drew Leder builds further on Merleau-Ponty's perspective of the lived body. In his book, *The Absent Body*, he introduces various concepts, including *inner body*. *Inner body* first and foremost refers to the body's internal organs, including how these organs can be regarded as more anonymous and unavailable compared to the *surface body*. Elaborating on these contrasting aspects, Leder notes that the “perceptual and expressive surface always rests upon a hidden base.” He further notes how the inner organs are for the most part “neither agents nor objects of sensibility.” Rather they constitute what can be described as “their own circuitry of vibrant, pulsing life” (Leder, 1990, pp. 64–65). Processes associated with the inner body—including the stomach and intestinal system, the endocrine and hormonal system, the circulatory system of the blood, and the respiratory system—have, in other words, some distinctive features compared to the surface body; features associated both with perception and movement. Despite these differences, Leder points to their chiasmic interrelatedness in a wide range of situations (Leder, 1990, pp. 40–56).

In the following pages, I elaborate on the particular methodology I have applied in this article. In particular, I highlight how hermeneutic phenomenology, as advocated by van Manen, proved to be a powerful tool for developing and analyzing the empirical material (van Manen, 1997). I also elaborate on my approach as a researcher—specifically, combining interviews with observation—and how the inter-subjective dimensions of this approach played a significant role in determining which findings were given priority.

## Combining interviews with observations

In recent years, scholars have noted the value of coupling in-depth interviews with participant observation. In combination, these two approaches provide more nuanced and complex insight into participants’ experiences than is available from research using only one of these approaches (Fangen, 2010; Patton, 2002; van Manen, 1997). Pursuing this line of thought Van Manen points out that the interview process usually involves an observing and sensitive researcher constantly reflecting on his own experiences as well as the participant's responses to questions being posed. Similarly, the close observer will usually engage in conversations and small talk with the participants under scrutiny in a variety of situations and settings (van Manen, 1997).

I decided to start as a participant observer. The experiences I acquired through this approach became significant during my subsequent interviews. Conducting individual in-depth interviews, which lasted approximately 1–1.5 h, I asked each of the women to elaborate on episodes or situations that I had witnessed in which she had been involved in one way or another, as well as for her comments on occurrences that had involved other participants, including me. As noted by van Manen, reflection on previous episodes or situations has a special value to researchers inquiring into lived experience. It gives the participant an opportunity to consider experiences that are “already passed” or “lived through” (1997, p. 10–13).

I contacted the women to arrange a second interview approximately 1 year after the first. During these follow-up interviews, I tried to pick up from where we had left off in our previous conversation, taking into consideration each woman's individual situation. In practice, this meant that I started the interview by asking how she was doing compared to the last time we had spoken. This question usually elicited a detailed response. Whereas some of the women brought up the topic of side effects and how these side effects affected their efforts to exercise on a regular basis, others emphasized their determination to continue exercising according to the interval principle. Still others explained that they had decided to drop out of the program. These various responses on the part of the women inspired me to ask follow-up questions that appeared relevant there and then. For example, when “Lilly” —introduced earlier in this article, mentioned that she had dropped out of the program before completing it, I encouraged her to elaborate on her decision providing me with vivid examples and anecdotes. Similarly, when “Lisa” emphasized her determination to continue exercising on a regular basis—I asked her to provide a detailed response as to what kind of exercise she was involved in after completing the rehabilitation program.

## Participant observation

I used participant observation as part of my research on women's experiences of group-based rehabilitation. By assuming a dual role as both participant and observer, the researcher can acquire first- as well as second-hand experiences of the settings and situations under study. According to van Manen, this approach—which he terms close observation—is likely to give the researcher experiential material that is different from what is available through interviews alone. Being present when intriguing incidents or episodes occur gives a researcher first-hand knowledge of these *anecdotes*, as opposed to learning about them solely through the accounts and interpretations of participants (van Manen, 1997, p. 29). Van Manen is equally clear, however, that the researcher needs to distance herself from the participant's life world. This distancing—or *hermeneutic alertness* as he terms it—should be regarded as an ongoing process in which the researcher “steps back” and “reflects” on the meanings of various situations and episodes (van Manen, 1997, p. 69). As such, close observation involves a delicate balancing act: getting as close as possible to the participant's life world, including the situations and settings she is involved in, while at the same time striving to maintain professional distance.

Bearing these aspects in mind, I conducted participant observation at the rehabilitation clinic where I had recruited the women. My ambition was to obtain a vivid picture of how the groups were organized and how the women responded to the various groups. In addition, I was hoping to gain insight into how they related to each other in the training sessions as well as to the health professionals running them.

Over a period of 3 months, I visited the clinic approximately once a week. My primary focus was on conducting participant observations in the surgery group. In practice, this meant that I joined the group during indoor training (eight sessions); and the Nordic walking group, which included participants from both the post-surgery group and the follow-up group (four sessions). I also spent time with the participants during session breaks, in the wardrobe before and after sessions, and in the reception area.^2^

Drawing on van Manen's recommendations concerning *close observation*, I took an active approach in the training sessions. When they played ball games, I joined one of the teams. During strength training, I tried to lift the same weight as my partner, even while trying to observe the other participants in the room. Similarly, during Nordic walking sessions I tried to accompany some of the participants while paying attention to the physiotherapists’ instructions concerning pulse, pace, and intensity. Although I did not become as tired, out of breath, and sweaty as some of the participants seemed to be, I experienced how exercising with them changed my focus from visual perception to bodily engagement. Being aware of my senses, sweating, and heartbeat gave me a closer connection to the women and their “reality” in this setting (van Manen, 1997).

My bodily sensations as participant observer—including my experiences of engaging in small talk with the women—provided me with first-hand knowledge of various aspects of the sessions, including the different atmosphere in each activity. In the Nordic walking sessions, for example, I chatted with the women in the rear of the group as we walked toward the hill where the (more intensive) interval session began. Laughter and small talk quickly ceased as the participants focused on climbing the hills. On the walk back down they were breathing heavily, some were completely out of breath. Each time they repeated the sequence the women became more exhausted. Some of them felt so dizzy or nauseous that they had to sit down for a while; others seemed determined to keep going.

As a participant observer my approach was to focus on the women who were included in the study. However, to avoid any questions that other participants might have concerning my presence (particularly in the mixed group sessions which also included non-surgical participants) at the start of each session I usually explained my position as a researcher, and emphasized that my focus first and foremost would be on the study participants. I never experienced any objections to my presence in any of the group sessions. However, I did find it challenging to focus my observations solely on participants in the study. Spontaneous situations involving study participants and other group members sometimes generated relevant data, particularly in the mixed Nordic walking group. Often, they involved newcomers and more experienced participants, or surgical and non-surgical participants. After one of these sessions, one of the non-surgical participants offered me a ride. Sitting in the car together with her and three other non-surgical participants, I felt excluded from participating in their conversation. My sense of being an outsider in this situation felt a bit strange, even uncomfortable, while at the same time interesting, as they were engaged in an animated discussion of their experiences with the interval training. In other words, it was hard to forget about certain situations, or pretend to set them aside, when I had actually participated in them. Other scholars have noted the challenges of observing rigid guidelines concerning what to observe and what not to observe. According to Fangen, it is nearly impossible to adhere to informed consent at all times and in all situations involving a variety of groups without jeopardizing the researcher's search for relevant data concerning the participants’ “reality” (Fangen, 2010, pp. 189–199). The findings in this article focus on the experiences of the women who were participating in the study. However, my observations of other participants in the groups have enhanced the contextual background and; I would argue, thereby enriched the analytical process.

### Participants

This article draws on the experiences of 11 women all of whom had undergone the gastric bypass procedure. The facility from which they were recruited was an inter-disciplinary clinic specializing in rehabilitation services for patients with problems related to obesity, including individuals undergoing weight loss surgery. Their program was inspired by the national guidelines for morbid obesity, and emphasized guidance and support for lifestyle changes. The group-based program, led by nutritionists and physiotherapists, included nutrition and training groups as well as discussions facilitated by a psychologist. The clinic received funding from the public health system and patients undergoing weight loss surgery had their costs covered during the 1-year program.

Most patients undergoing weight loss surgery were enrolled in a 1-year program, consisting of a 3-month introductory phase and a 9-month follow-up phase. During the introductory phase, the patients were enrolled in a surgery group with 10–14 participants. This group took part in training sessions every week, as well as dietary sessions every second week under close supervision by physiotherapists and nutritionists. During the follow-up phase, participants in the surgery group were mixed together with other patients in the clinic, primarily patients with obesity-related problems enrolled in the clinic's conservative lifestyle program. The mixed group was roughly twice the size of the surgery group, typically 20–30 people in different stages of the rehabilitation process. Participants in this mixed group were also guided and supervised by nutritionists and physiotherapists.

Although the rehabilitation process was group-based, participants began and completed the program at different times, so that each group contained both newcomers and more experienced patients and they engaged in the same activities. For example, because patients usually had to wait for months to get into the surgery group some participants had undergone surgery fairly recently, others a long time before, and still others had not had it yet. Because of long waiting lists for weight-loss surgery funded by the public health system, some patients had to wait 1 or even 2 years for their operation. Members of the follow-up group were also in different stages of their rehabilitation process. Some had recently been transferred from the surgery group to the follow-up group, some were halfway through the 9-month follow-up phase, and others had nearly completed it.

I began the process of recruiting participants after receiving approval from the research ethics committee of medicine in Norway as well as the clinic's management. When I presented the study in the surgery group, four of them showed immediate interest in participating. Additionally, seven women in the follow-up group agreed to participate. The inclusion of participants in different stages of their rehabilitation process and from two different group settings provided an opportunity to obtain more varied data on women's experiences in the program.

The women ranged in age from 30 to 55 years. Most of them were working full- or part-time. Four had university or college degrees. Two had enrolled in college or university as part of a rehabilitation process, following a long period of absence due to illness from their previous job. Five of the women had completed a high school vocational program. Only three of the women were single; the rest were in a relationship or married. The majority of the women had children, all of whom had been born prior to the surgery.

As noted previously, the dates of participants’ surgery ranged considerably. At the time of the first interview, most of the participants had undergone the gastric bypass operation within the previous 8–12 months. I contacted the women approximately 1 year later for a follow-up interview. Two did not respond. Another was ill at the time so that we decided to postpone her second interview. Table I provides an overview of the women's age and BMI prior to their surgery, and time elapsed between their surgery and the first interview. To safeguard confidentiality the participants are designated with a number.

**Table I:** Participants.

Participant	First interview	Follow-up interview	BMI	Age
1	About 1 year	Yes	54	35
2	About 10–11 months	No (Illness)	42	55
3	About 1 year	No (Did not return my calls)	Did not recall	34
4	About 1 year	Yes	About 65	40
5	About 1 year	Yes	Did not recall	43
6	About 8 months	Yes	About 54	47
7	About 9 months	Yes	About 42–43	32
8	About 9–10 months	No (Did not return my calls)	Did not recall	32
9	About 1 year	Yes	49	30
10	About 1 year	Yes	42–43	47
11	About 16 months	Yes	About 46	44

### Ethics

This study was approved by the Norwegian Regional Committee for Medical and Health Research Ethics. Information about the study was provided to potential participants along with the letter asking if they would be willing to join the study. The letter emphasized that participation was voluntary, and that they could withdraw from the study at any time. They were also assured that any published material would not include any of their personal information, such as their name or other details that could establish their identity (Kvale & Brinkman, 2009; Ruyter, 2007). Once participants had signed the informed consent form, I took pains to preserve their confidentiality, utilizing fictitious names in the article and omitting details such as age, social status, family background, and professional background that could identify them.

## Analysis

Deriving the most benefit from combining interviews with participant observation required selecting and interpreting the varied findings—in the sense of determining which episodes, topics, or phrases were significant to my research question. Selecting and interpreting these findings took place both in the moment and later while I was writing and re-writing descriptions of what I had witnessed (van Manen, 1997). This ongoing writing process is at the heart of the phenomenological-hermeneutic approach, serving as the essential mechanism for discovering analytical points often only vaguely grasped during the actual observation or interview. As van Manen points out; “Sometimes the best anecdotes are re-collected as one tries to make sense of things that somehow seem interesting now, in hindsight” (1997, p. 69). In other words, reflecting on episodes, topics, or phrases in retrospect generates not only valuable interpretations, but also important new questions.

When I started the process of writing this article, I began by perusing my notes, carefully searching for details that could be of relevance. Reading them I found myself recalling “new” details that I had not written down. In the process of elaborating on what I had originally put down and writing and rewriting my explorations of various themes that emerged, I expanded the empirical material to include written recollections, as well as embodied recollections stimulated by my analytical process. This analytical approach is consistent with van Manen's ideas on how empirical material should develop over time (van Manen, 1997).

I followed a similar path with empirical material developed through the interview process. Listening to the tape-recorded interviews again allowed me to relive the sessions. For example, I could *visualize* the women's facial expressions as they elaborated on their aversive experiences of pushing themselves while at the same time sensing dizziness and fatigue. Moreover, my own sensations during these sessions suddenly “came back,” including my sense of embarrassment over listening to the women's detailed elaborations over their annoyance and anger against the physiotherapists pushing them to walk faster without revealing my negative thoughts. These embodied recollections—coupled with the women's ambivalent experiences toward the interval training—enabled me to formulate specific questions concerning the empirical material: How did the women express their ambivalence? How did they relate to this ambivalence? How did the women account for their decision to drop out of or complete the program? Posing such questions as I studied the written transcripts enabled me to compare the women's experiences systematically.

In the process of looking as closely as possible at the women's own experiences, meaning was primarily confined to their *self-understanding*. As advocated by Kvale and Brinkman, this level of analysis involved formulating what the women themselves understood to be the meaning of their experiences (2009, p. 214). In addition, the analysis involved reformulating the women's self-understanding in the context of common-sense understanding (Kvale & Brinkman, 2009, p. 215). Discussions with colleagues were fruitful in this process helping me establish a hermeneutic distance from the participants. Distancing myself from the women's accounts enabled me to interpret their experiences in a new light and develop a more nuanced, common-sense understanding of the empirical material. Finally, theoretical concepts and ideas enabled me to regard women's experiences as unique and embodied, and at the same time interrelated with the women's larger sociocultural context, and their relationships with others.

It should be noted that these interpretations involved in a spiral, as advocated by van Manen (1997). In his hermeneutic-phenomenological approach, moving back and forth between parts of the material and the whole through the interrelated processes of reading, listening, discussing, and writing creates the potential for a continuously developing understanding of meaning.

## Findings

During the analytical process two principal themes emerged: *Pushing one's tolerance limits* and *Rebutting the “*quick fix” *fallacy*. In different (though interconnected) ways, these two themes encompass an ongoing change in these women's lives—namely, their efforts to establish new exercise habits while working hard to rebut the popular belief that weight loss surgery is a quick fix.

### Pushing one's tolerance limits

The significance of pushing one's tolerance limits emerged as a recurring aspect of the Nordic walking sessions. This mixed group consisted of newcomers and more experienced participants. Halfway through the circuit the physiotherapists would organize hill intervals, during which the participants were instructed to push themselves to the max, or nearly to the max.

During their first weeks as newcomers, the women in the study found pushing their tolerance limits during interval sessions uncomfortable and extremely challenging. “I thought I would die, I really hated it,” Mary commented. Similarly, Lisa described these sessions as “fucking awful,” adding that she could not envision “doing this” on her own.

Even though the women tried their best to expend extra effort after years of engaging in far less physical activity, they felt pressure to push themselves even more. Helena elaborated on this challenge:

That's part of the deal, I guess. They organize the sessions, particularly the Nordic Walking sessions, so that the physiotherapists …. … they focus on pushing you … they try to make you push yourself even more than you think you can …. “Come on, try a bit harder, and a bit harder …” mmmm.

Although the women found it challenging and uncomfortable to keep pushing themselves, as well as to be pushed by the physiotherapists supervising these sessions, only a few negotiated with themselves on whether to quit or continue for the remaining weeks of the program. The majority accepted the premise that pushing beyond one's comfort zone was necessary to ensure a successful result. “The surgery is just a tool, you know,” Elisabeth declared, “You have to do the job yourself.” Heidi's biggest fear was that after the surgery she would regain weight. To prevent this, she was willing to endure a lot of sweat and tears. In a similar vein, Helena noted that she kept pushing herself despite bodily discomfort because the alternative was much worse—that is, the prospect of regaining weight. Moreover, given her ongoing struggle with food and eating (exemplified in interview descriptions of binge eating in situations where she felt stressed and had an overwhelming urge to “eat and eat”), Helena considered it vital to complete the training sessions. Indeed, she regarded the interval sessions as a form of protection:

When everything else is a challenge and I can't stop myself from overeating … the training serves as my buffer …. I just have to keep it up.

Charlene, on the other hand, found these training sessions hard to endure and dropped out halfway through the program:

I just couldn't take the pressure any more. It was a bit too much, so to speak. It's nice to be pushed, in a way, but if you had a bad day and couldn't push yourself as much as was expected of you … “Come on Charlene, come on.” So I figured I had to take control in my own way – be in charge of my own training, so to speak. Some days it was a challenge just getting there … to the clinic … you know …. That was a victory in itself for me. So when pressure was piled on top of that … well … I just couldn't take it anymore …. Pressure is okay once in a while … but not all the time … so I decided to quit ….

As the months progressed, the women who continued to participate in the Nordic walking sessions experienced dramatic weight loss. And as they lost more and more weight, they became more and more enthusiastic. Their enthusiasm was closely intertwined with their progress in the mixed group sessions, particularly by the change in their position from lagging in the back of the group to being able to walk in the front.

These women stressed their determination to continue pushing themselves and ensure that their surgery would lead to long-term weight loss. In contrast, two of the women detested the interval sessions to such a degree that they invented white lies to avoid them. This enabled them to continue in the program rather than drop out permanently.

### Rebutting the *“quick fix”* fallacy

Regardless of whether women dropped out of the clinic's year-long program or decided to complete it, they shared the view that undergoing weight loss surgery was anything but a quick fix. It wasn't necessary to emphasize this in conversations with other participants in their group—they were all in the same situation. “I feel at home in this group,” Cathy commented earnestly, “We're in the same situation, in a way, and we understand each other.” In a similar vein, Stine noted how nice it was to exercise with others who were in the same situation and not have to justify her decision to undergo surgery. In particular, she emphasized how fatigue kept her from pushing herself the way non-surgical participants could.

The women spoke enthusiastically about the warm, caring, atmosphere in their small surgery group, where they felt everyone was in the same situation. They supported each other when anyone was having problems with side effects, and encouraged each other's efforts to build exercise routines, as well as change eating habits. Trudy, for example, volunteered that when she was desperately in need of someone to talk to, she knew whom to call—suggesting that she felt the women she had met at the clinic were more understanding and supportive than other members of her social network.

The feeling of belongingness the women enjoyed in their surgical group contrasted sharply with their sense of alienation in the mixed Nordic walking sessions. As a participant observer, I noticed that non-surgical patients rarely talked to the women who had undergone weight loss surgery. However, it wasn't until I commented on this during the interviews that the women themselves revealed their efforts to rebut the popular notion of weight loss surgery as a quick fix. In this context, they would particularly articulate the significance of performing exercise routines on equal terms with non-surgical patients. Here is how Lilly recalled one episode:

You could tell by their looks and how they talked when I had lost 30 kg. “Oh, it's so easy for you.” So I felt I had to tell them, “Look at me! I have to exercise, just like you ….”

In addition to verbally rebutting the quick-fix fallacy, the women did their best to keep up with the non-surgical participants. Indeed, even when troubled by intestinal problems or fatigue, they refrained as well as they could from voicing these difficulties in the mixed group sessions. According to the women, they reached a turning point when they finally managed to walk at the head of the group. Indeed, their ability to walk in front made it clear to the entire group, as well as to themselves, that their efforts had paid off. “It was fun,” recalled Elisabeth. “It was wonderful … I really enjoyed that.”

This is not to say that Elisabeth enjoyed walking at the head of the group during the remaining months of her 1-year program. After 6 months, fatigue became a major problem for her:

Having enjoyed this … all of a sudden you couldn't keep up the pace any more …. You felt empty, in a sense … out of energy … completely out of energy. I think it started in November or December and then, suddenly, a kind of flat battery … absolutely nothing more to give …. You run completely out of energy.

In the ensuing months, Elisabeth had to drop out of the interval sessions. However, she returned when her fatigue became less debilitating. Stine, on the other hand, was determined to keep going despite experiencing increasingly problematic side effects. Lagging in the back of the group, she could sense the gaze of others as they passed her. Ultimately, she found these sessions so uncomfortable and exhausting that she dropped out of the program entirely. Gradually, Stine established her own routine, taking daily walks at a slower pace. Walking accompanied only by her dog, she was able to determine the distance and intensity on her own, and was eventually able to take longer hikes in the woods without feeling exhausted or out of sorts. These self-guided excursions gave her a sense of freedom and independence that she had not experienced in the training group. Bibi also dropped out of the rehabilitation program and developed alternative ways of exercising that she felt “suited her personality better.” By personality, she meant her characteristic of “never being an exercise type,” and a wish to be active in a manner better suited to her needs and preferences. She particularly enjoyed water gymnastics, which she had actively engaged in prior to her surgery. After dropping out of the clinic program, Bibi resumed water gymnastics in her hometown.

The women who completed the 1-year program at the clinic emphasized that the discipline required in the group sessions enhanced their sense of being in control of their situation, particularly of their weight loss efforts. Lisa explained it this way:

My huge fear is that this is going to come apart somehow. I've invested such an awful lot in it … For me, working out is a must … You receive a tool when you go through that surgery … but in a way, you have to come to grips with everything else as well …. My life is organized so that I'm able to get my sessions in during the week.

In a similar vein, Cathy emphasized how her dramatic weight loss—combined with her efforts in the interval sessions at the clinic—had increased her self-confidence. One year after enrolling in the program, she enjoyed exercising on equal terms with her friends at a local fitness center—without feeling clumsy or attracting unwanted attention. Prior to the surgery, the gaze of others had prevented her from exercising in ordinary fitness centers. Moreover, to escape the gaze of work colleagues if she were unable to keep up during group activities, she had avoided after-hours social events such as trips:

Had someone told me that I would actually exercise with ordinary women on equal terms, I would not have taken that seriously. No way, not me …. So I feel kind of proud of myself and what I have accomplished … through hard work.

As these comments illustrate, Cathy attributed her success primarily to her own ongoing efforts, rather than to the surgery itself. These efforts, she felt, had ensured a successful outcome, an outcome that she was proud of, and that was enabling her to socialize in new ways that she had previously considered unavailable.

## Discussion

The findings highlight how being part of a group-based program triggered the women to get started with regular exercise after years of living restricted lives in terms of movement and physical activity. During their initial months in the rehabilitation program, the women tried their best to adjust to the group-based sessions, including the significance of pushing themselves. At the same time, they tried to tone down the effects of the surgery, including problematic side effects. Particularly in the mixed Nordic walking sessions, the women strove to exercise on equal terms with non-surgical patients, rebutting the notion of weight loss surgery as a quick fix explicitly and implicitly. This is understandable, considering the controversies and stigmas that a weight loss surgery connotes. Post-surgical patients who share their stories in public, for example, tend to be met with critical inquiries concerning their choice of surgery. The rhetoric and positioning of professionals engaging in public debates, suggest conflicting views as to whether obesity should be understood as a disease, a lifestyle problem, or both. Moreover, there is controversy with regard to how obesity should be treated (Fox, Ward, & O'Rourke, 2005; Hofmann, 2010; Lupton, 2013; Svenaeus, 2013; Rugseth, 2011; Throsby, 2009). During the past 4–5 years, Norwegian professionals representing different backgrounds and specialties have increasingly engaged in this debate. Take, for example, general physician Ramstad's newspaper chronicle in which he compares weight loss surgery with *lobotomy*. His point in using the term lobotomy is to emphasize that surgery is not capable of curing the patient's problems. Rather than destroying well-functioning and healthy intestines, he advices surgeons to concentrate on patients’ lifestyle problems, and encourage them to exercise more and eat healthier food.^3^ Similar views have been brought forward by Kari Jacquesson a profiled exercise expert in Norway.^4^ Rather than acknowledging surgery as an effective treatment against obesity, she sees it as *a quick fix* for those not willing to make radical changes in their lifestyles.^5^ Given their expert status in the public debate, one could therefore argue that these experts’ approach and rhetoric serve to perpetuate an impression of surgery being an easy solution compared with traditional ways to lose weight. Accordingly, stigmas and popular assumptions may be reinforced by those who choose this form for treatment.

The women's efforts to push themselves while trying their best to tone down the problematic aspects of the surgery can further be elaborated on through Merleau-Ponty's ideas about the intertwinement of body and world. Although the boundaries between body and world are clear-cut from an objective point of view, they are generally experienced as quite ambiguous. Following this line of thought, Swedish philosopher Lisa Käll acknowledges Merleau-Ponty's image of the hard, sticky honey almost impossible to remove from the skin:

It comes apart as soon as it has been given a particular shape and, what is more, it reverses the roles by grasping the hands of whoever would take hold of it. The living, exploring hand which thought it could master this thing instead discovers that it is embroiled in a sticky external object. (Käll, 2006, p. 224)

To visualize the stickiness of the honey is, according to Käll, to pinpoint a situational relationship between the individual and her surroundings. Whereas the stickiness of the honey can be washed away with soap and water, the stickiness of other peoples’ attitudes and assumptions may be more difficult to get rid of. Yet, as individuals embedded in the world, we are compelled to relate to our surroundings (Käll, 2006, pp. 64–88). As for the women I interviewed, those who were the most bothered by side effects expressed ambivalence as to keep pushing themselves and eventually decided to drop out of the group. In accounting for their decision to drop out, stigmas and feelings of not fitting in the mixed group setting played a crucial role, closely intertwined with their experiences of not being able to push themselves according to the program's intentions. In particular, problems of energy loss and fatigue was increasingly sensed in the entire body and made it difficult to exercise on equal terms with the others. These women's experiences actualize Leder's perspective of the *inner* body. More specifically, he points to the viscera's connection to the body's hormone regulation, and that changes in this connection might have a significant impact on an individual's sense of being in equilibrium (Leder, 1990). Hence, when interval training is performed by individuals in such a state, there is a chance that their body's homeostasis is inflected in a more intense manner compared to activities of lower intensity. Supporting this line of argument, the women elaborated on their eagerness to engage in low intensity activities, such as walking at their own pace or water gymnastics. Indeed, they emphasized that these activities made them feel well and comfortable as opposed to the interval training in the program they dropped out of. The anthology *Meaning and Sports* thematizes the significance of a strong inner drive regarding the exercise or activity engaged in (Steen-Johansen & Neuman, 2009). This may constitute a feeling of euphoria upon entering into the flow and rhythm which makes an activity like downhill skiing so enthralling and it may be recognized in the feeling of floating into the music and oneself when one has acquired the necessary skills and aerobics become a prized free space (Loland, 2009; Steen-Johansen, 2009). Thus, one may forget one's worries and bodily complaints and concentrate on enjoying the here and now situation. Practicing long walks at one's own pace or water gymnastics may, in other words, be experienced as meaningful and thus activate an inner drive which motivates one to continue with this form of exercise in the longer run.

As for those women who emphasized their determination to keep up with the group training, *fear of weight regain* intertwined with their efforts to keep pushing themselves. At the same time, the findings suggest that while they were in the program, the women worked hard to comply with the health professionals’ instructions. This raises the question of how much freedom of choice the women actually had in the group-based rehabilitation setting. Focusing on a pre-surgical lifestyle program, Knutsen found that participants felt constrained to position themselves as obedient to the advice and instructions of the health professionals. Participants who expressed criticism, for example by refusing to comply with some of the advice concerning dietary changes, risked having their forthcoming surgery postponed, or worse, cancelled. Having passed initial screening and waiting to have their surgery date scheduled, while at the same time having to participate in the hospital's compulsory preparation program, a lot was obviously at stake for them. According to Knutsen, participants’ loyalty and compliance during group discussions can thus be understood as a strategic means of positioning themselves as suitable for the forthcoming weight loss surgery (Knutsen, 2012). As the women in my study had already undergone the gastric bypass procedure, their situation differed from that of the participants in Knutsen's study. After their surgery, the women decided whether they wished to join post-surgical rehabilitation programs, and what kind of program to join. However, it would be misleading to term their participation entirely voluntary. The ambivalence some of the women expressed about continuing to participate in the rehabilitation program despite experiencing unpleasant side effects and bodily discomfort suggests that a lot was at stake. Perhaps they feared ending up as unsuccessful participants? Up until now the literature has essentially divided respondents into successful and unsuccessful patients; to explain variability in long-term results, researchers have increasingly emphasized *nonsurgical* factors (Bond et al., 2010; Fried et al., 2008; Hofsø et al., 2011; Kristinsson, 2008; Lier, Biringer, Bjørvik, & Rosenvinge, 2012a; Lier, Biringer, Stubhaug, & Tangen, 2012b; Snyder et al., 2009; Steffen, Potoczna, Bieri, & Horber, 2009). Since the beginning of this century, the crucial role of lifestyle changes in ensuring successful outcomes have also been emphasized in national guidelines issued by public health authorities in most Western countries. In line with previous findings, undergoing gastric bypass surgery can be considered a turning point in terms of physical activity and exercise (Groven, Råheim & Engelsrud, 2013b). However, only follow-up studies can determine whether the women will continue to engage in regular exercise in the years to come.

While many scholars have drawn on Leder's ideas concerning bodily dys-appearance, few scholars have explored situations in which the body is experienced in more harmonic and positive ways.^6^ Considering its deleterious and troublesome aspects, dys-appearance demands the attention and response not only of the individual herself but also of her health care professionals. These professionals try various medical treatments to soften bodily dys-appearance and achieve bodily dis-appearance for patients, to paraphrase Kristin Zeiler (2010, p. 324). Building on the perspectives of Leder and Merleau-Ponty, she argues for the need to explore nuances in bodily self-awareness, and how the body may appear differently to the person in different situations (Zeiler, 2010). The women I interviewed emphasized that they experienced daily and leisure activities as less exhausting after they had lost 20, 30, 40 or 50 kg. Obstacles they had associated with participating became less significant. The women's accounts also suggest, however, that side effects and bodily complaints did not disappear. In particular, those who struggled with fatigue and intestinal problems felt compelled to limit their activities for longer or shorter periods. Taken together, these nuances in bodily awareness reveal the challenges of categorizing patients as successful or unsuccessful based primarily on changes in their lifestyle. Although such categories may be useful in identifying significant patterns prior to and following weight loss surgery, such as changes in activity level, they do not illuminate the ways in which patients go about changing their activity and exercise habits or the complexity of these changes. A phenomenological approach has enabled me to discern variation, ambivalence and complexities in women's efforts to become more active following gastric bypass surgery. Hopefully, these insights can contribute to a debate regarding the validity of non-surgical factors in accounting for what determines whether a patient's surgery could be considered “successful” or not.

## Methodological considerations

As for methodological considerations, my combination of participant observation with individual in-depth interviews provided nuanced and complex insight into the women's experiences of participating in group-based interval training. Moreover, given the fact that all of the women had undergone the same surgical procedure, namely the gastric bypass surgery, insight into the challenges associated with this procedure in combination with interval training was also touched upon.

Additionally, conducting follow-up interviews could be considered a methodological strength. In the follow-up interviews the women talked about their efforts to change exercise habits over time, as well as elaborating on their experiences of dropping out or completing the rehabilitation program. In doing so, I was able to get in-depth insight into the challenges of mixed group training for women having undergone a gastric bypass procedure.

Unfortunately, two of the women in the study did not respond to my invitation for a second interview, and a third was ill. Ethical concerns prevented me from pressuring these women into granting a second interview. One could argue that this is a limitation of the study, given that the long-term experiences of these women are not reflected in the findings.

Since 10 of the 11 women included in the study were ethnically Norwegian, their homogeneity could be considered a limitation in terms of transferability. However, it should be noted that these 10 women varied considerably in terms of age, educational background, and social status. Another potential limitation is that all of the participants were recruited from one rehabilitation program, and both interval training and Nordic walking training can be organized in various ways. However, the study was not intended to reach statistical conclusions.

It could also be argued that my own pre-conceptions might have affected the findings, given my background as a physiotherapist with no clinical experience of patients undergoing weight loss surgery, as well as my background as a researcher whose work is informed by phenomenology. I would argue that these factors should not be regarded as limitations. As Linda Finlay has argued, the researcher is a central figure who influences the collection, selection and interpretation of data. To ensure trustworthiness, she or he should therefore reflect on how subjective as well as intersubjective elements have influenced the findings (Finlay, 2002, p. 531). In this regard, I made reflection on my approach an integral part of my research, both during the data collection process and after I had developed more distance from it. While taking part in the training sessions, I reflected on various questions: How did my presence in the group affect the participants? How should I behave? What could I say and not say?

To avoid making critical comments or being associated with the physiotherapists running the sessions, I emphasized my role as a researcher and played down my educational background as a physiotherapist. In addition, I positioned myself in the back of the Nordic walking group so that I would not be associated with the fittest and best-trained participants. Through these efforts, I was able to create a sense of trust that became invaluable during the interview process. I conducted all of the interviews personally and then transcribed them myself. This encouraged the women to speak candidly about their experience of the training, without fear that sharing their personal reflections might undermine their participation in the program, their relationships with the physiotherapists in charge of the program, and so on. Accordingly, when some of them expressed ambivalence about aspects of the program during the interviews and sometimes even criticisms, I would respond by encouraging them to speak as freely as they wished and affirm that their personal reflections were both interesting and worthy of attention. On occasions when my background as a physiotherapist “came up”, I consistently reiterated that I was conducting research, and was not employed as a physiotherapist at the clinic.

During the analytical process I found it useful to discuss preliminary themes and analytical points with researchers representing a variety of backgrounds.

Finally, I have tried my best to increase the trustworthiness of the findings by citing all 11 women who participated in the study. This showcases the variations among them and contributes to a nuanced picture of women's experiences of group-based interval training following weight-loss surgery.

## Concluding remarks

Based on my empirical findings, I find it hard to decide who the “successful” participants are. Are they those who go about changing their exercise habits in conformity with the guidance and recommendations of health professionals? Those who challenge post-surgical guidelines and norms and seek out activities appropriate to their own needs and desires? Those who manage to be active on their own despite experiencing problematic side effects, complications and bodily complaints? Those who feel the need to slow down and be less active for shorter or longer periods? These questions point to the need of acknowledging patients’ own experience to determine how “successful” they are after surgery. In doing so, this study has given greater recognition and significance to the intertwining of surgical and non-surgical factors than is customary in the literature that dominates today's debate concerning lifestyle changes following weight loss surgery.

Based on this intertwinement of surgical and non-surgical factors, patients undergoing weight loss surgery should be given more room for individual adjustment, choice and co-determination and be encouraged to trust their own bodily sensations during group-based interval training. Such an approach implies a mutual relationship between patient and physiotherapist, which allows exploration of what kind of activities and what intensity are best suited to individual needs and desires. Besides, it implies that the biomedical perspective on standardized and “effective” group training—which seems to dominate today's rehabilitation programs for morbidly obese patients needs to be supplemented with a more experience-based perspective on the body.

Hopefully, the article's insights into these matters will initiate a discussion of ways to consider patient preferences and needs in post-surgical rehabilitation programs. I believe it is crucial for physiotherapists to become more aware of the complexity of challenges associated with changing activity habits, and the variations among women experiencing these changes following gastric bypass surgery.

In future research, exploring ways of combining group-based rehabilitation with individualized supervision and guidance could be particularly fruitful. A longitudinal study based on several interviews and observations over a period of more than 5 years could be particularly useful in developing more insight into the needs of individual patients. It could also identify critical phases in the process of adjusting to a changed and changing body while attempting to alter activity habits. In addition, it would be extremely useful to explore the life situation of the women who participated in this study over time. Of particular interest would be an inquiry into their exercise habits 5, 10, and 15 years following their surgery.

## Footnotes

1 The importance of changing one's lifestyle after weight loss surgery as a means of ensuring “successful” outcomes (in terms of weight loss and quality of life) is also emphasized in the national guidelines—issued by the Norwegian Ministry of Health and Care Services (Health promotion and treatment of morbid obesity within the specialized health service).

2 Nordic walking (also called pole walking) is brisk walking with specially designed poles. Nordic walking involves applying force to the poles with each stride. Accordingly, the use of walking poles can potentially result in increased oxygen consumption and as well as increased caloric expenditure compared to regular walking. Indeed, Nordic walking has been estimated as producing up to a 46% increase in energy consumption, compared to walking without poles. Pole walking has become a popular form of training in Scandinavia, both for healthy individuals, and patients with chronic illness, including morbid obese patients participating in lifestyle programs (Figard-Fabre, Fabre, Leonardi, & Schena, 2010; Jönsson & Johansson, 2009; http://en.wikipedia.org/wiki/Nordic_walking).

3
http://www.adressa.no/meninger/article1725408.ece

4
http://karijaquesson.blogg.no/1348497844_jrgen_foss_er_talsman.html

5
http://www.vg.no/helse/artikkel.php?artid=10025416

6 See Duesund & Skårderud (2003), “Use the body and forget the body: Treating anorexia nervosa with adapted physical activity”; and Gimlin (2006), “Cosmetic surgery as a response to bodily dys-appearance.”
