# Corrigendum

**DOI:** 10.3402/qhw.v9.25927

**Published:** 2014-09-05

Regarding the paper titled: ‘‘They think surgery is just a quick fix’’ by Karen Synne Groven.

Published in *International Journal of Qualitative Studies on Health and Well-being*, 11 July 2014. Citation: Int J Qualitative Stud Health Well-being 2014, **9**: 24378 - http://dx.doi.org/10.3402/qhw.v9.24378

On page 2, second column second paragraph (“Both the risk of regaining weight …”), the sentence currently reads: “However, we also found that the women's changing habits were intimately intertwined with their changed and changing bodies, including side-effects and changes in their bodily capacity (Groven et al., 2013).”

This sentence should read: “However, we also found that the women's changing habits were intimately intertwined with their changed and changing bodies, including side-effects and changes in their bodily capacity (Groven, Råheim & Engelsrud, 2013a).”

On page 11, first column first paragraph (“As for those women who emphasized …”), the sentence currently reads: “In line with previous findings, undergoing gastric bypass surgery can be considered a turning point in terms of physical activity and exercise (Groven, Råheim & Engelsrud, 2013).”

This sentence should read: “In line with previous findings, undergoing gastric bypass surgery can be considered a turning point in terms of physical activity and exercise (Groven, Råheim & Engelsrud, 2013b).”

One reference was missing from the article. The references have now been supplemented with: “Groven, K. S., Råheim, M., & Engelsrud, G. (2013a). Changing bodies, changing habits: Women's experiences of intervall training following gastric bypass surgery. *Health Care for Women International*. DOI: 10.1080/07399332.2013.794465.”

The reference in page 13 should read: “Groven, K. S., Råheim, M., & Engelsrud, G. (2013b). Disappearence and dys-appearance anew: Living with excess skin and intestinal changes following weight loss surgery. Medicine, Health Care and Philosophy. DOI: 10.1007/s11019-012-9397-5.”

